# Supplementary material for: Pituitary Tumor Transforming Gene 1 Orchestrates Gene Regulatory Variation in Mouse Ventral Midbrain During Aging
Source: Front Genet. 2020 Sep 23;11:566734. doi: 10.3389/fgene.2020.566734 (PMC7538689; doi:10.3389/fgene.2020.566734)
Supplement: Supplementary file 4 [file Data_Sheet_1.PDF]

Pituitary Tumor Transforming Gene 1 orchestrates gene regulatory variation in mouse ventral midbrain during aging

Yujuan Gui#, Mélanie H. Thomas#, Pierre Garcia, Mona Karout, Rashi Halder, Alessandro Michelucci, Heike Kollmus, Cuiqi Zhou, Shlomo Melmed, Klaus Schughart, Rudi Balling, Michel Mittelbronn, Joseph H. Nadeau, Robert W. Williams, Thomas Sauter, Manuel Buttini*, Lasse Sinkkonen*

Supplementary Information

Supplementary Figures

Supplementary Figure S1. RT-PCR measurements of *Pttg1* expression in isolated midbrains are consistent with the RNA-seq results.

A. *Pttg1* expression measured by RT-PCR is consistent with the RNA-seq data across the three strains. Expression levels are presented relative to *Gapdh*. Two-sided Student's t-test was used for statistical testing. *=p<0.05.

B. *Pttg1* expression measured by RT-PCR is consistent with the RNA-seq data across the 3 months old *Pttg1*$^{+/+}$, *Pttg1*$^{+/-}$, *Pttg1*$^{-/-}$, and 9-13 months old *Pttg1*$^{-/-}$ mice. Expression levels are presented relative to *Rpl13a*. Two-sided Student's t-test was used for statistical testing. *=p<0.05.

Supplementary Figure S1

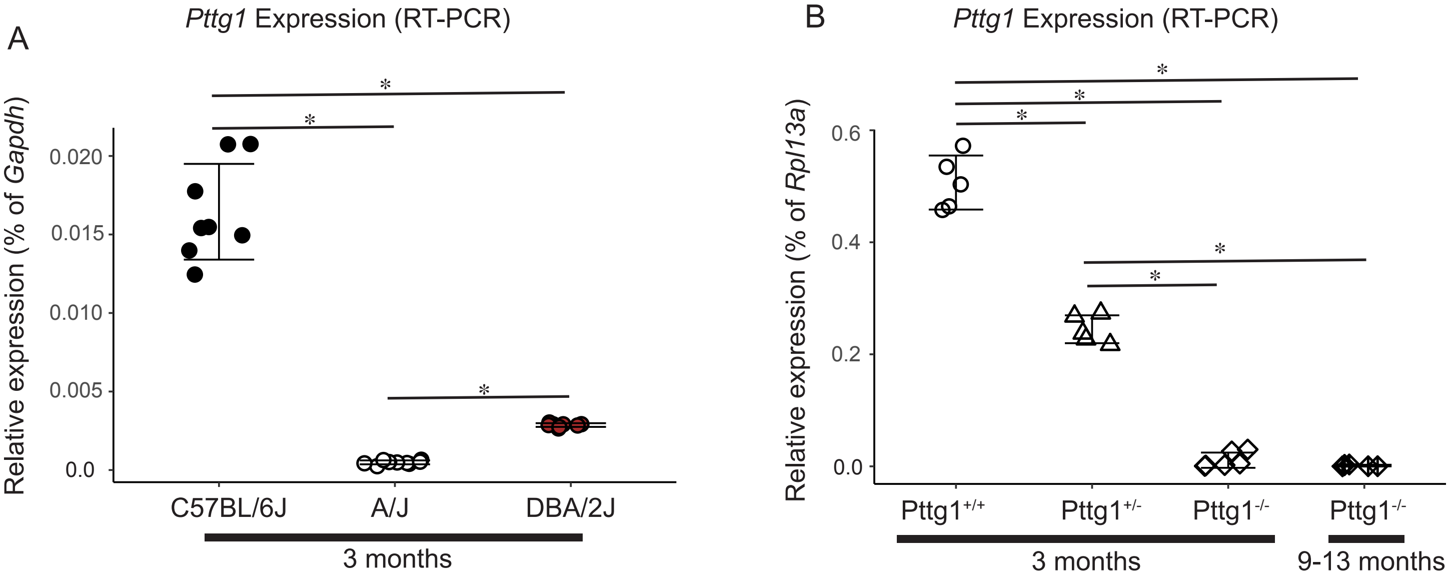

Supplementary Tables

Supplementary Table S1. **Primer sequences used in the study.**

Gene	Forward primer (5' – 3')	Reverse primer (5' – 3')
Pttg1	TCAAGGTCGGCTGTTTTGGT	AGTTGCCGAAAAGCCTATGAAG
Rpl13a	TGGTCCCTGCTGCTCTCA	CCCCAGGTAAGCAAACTTTCT
Gapdh	TGCGACTTCAACAGCAACTC	CTGCTCAGTGTCCTTGCTG

Supplementary Table S2. DEGs (FDR < 0.05, log2FC > 1) from three comparisons in Figure 2A. The base mean, log2FC, and FDR are reported for each gene in each comparison: A/J vs. C57BL/6J: 1145 genes; DBA/2J vs. A/J: 1039 genes; DBA/2J vs. C57BL6/J: 1251 genes.

Supplementary Table S3. DEGs (FDR < 0.05, log2FC > 1) shared by at least two comparisons in Figure 2B. The base mean, log2FC, and FDR are reported for each gene in each comparison: A/J vs. C57BL/6J: 853 genes; DBA/2J vs. A/J: 804 genes; DBA/2J vs. C57BL6/J: 980 genes.

Supplementary Table S4. DEGs (FDR < 0.05, log2FC > 2.5) from 3 months old vs. 9 months old mice. The DEGs from C57BL/6J A/J, DBA/2J and *Pttg1*$^{-/-}$ mice are shown on individual worksheets. The base mean, log2FC, and FDR are reported for each gene in each comparison.
